# Phosphonic Acids as Corrosion Inhibitors and Adhesion Promoters for Organic Coatings and Bronze

**DOI:** 10.3390/ma17153710

**Published:** 2024-07-26

**Authors:** Dajana Mikić, Floren Radovanović-Perić, Helena Otmačić Ćurković

**Affiliations:** Research Laboratory for Corrosion Engineering and Surface Protection, University of Zagreb Faculty of Chemical Engineering and Technology, Marulićev trg 19, 10000 Zagreb, Croatia; dmikic@fkit.unizg.hr (D.M.); fradovano@fkit.unizg.hr (F.R.-P.)

**Keywords:** adhesion promoters, bronze, corrosion protection, cultural heritage, phosphonic acid

## Abstract

Currently used organic coatings for the protection of bronze sculptures have a relatively short lifespan as a consequence of strict requirements of conservation ethics, which limit the selection of coatings. For that reason, enhancement of the corrosion protection level and durability of appropriate coatings is needed. The aim of this work was to examine if corrosion protection of bronze by selected acrylic and polyurethane coatings could be improved by using two phosphonic acids, 16-phosphonohexadecanoic acid (COOH-PA) and 12-aminododecylphosphonic acid (NH_2_-PA). Electrochemical measurements (linear polarization and electrochemical impedance spectroscopy, EIS) were performed to gain an insight into the influence of these phosphonic acids on the performance of the coatings during a two-week exposure to artificial acid rain and a three-month outdoor exposure. Besides the influence on the corrosion protection level, the influence on the coating adhesion was examined as well. A pull-off test clearly confirmed that the studied phosphonic acids act as adhesion promoters of both polyurethane and acrylic coatings, while electrochemical studies revealed improvements in corrosion protection levels, especially in the case of the acrylic coating Paraloid B72.

## 1. Introduction

Outdoor sculptures are mainly made of bronze. Despite its good corrosion properties, bronze is still susceptible to decay in a polluted urban atmosphere [1]. To slow this process, transparent organic coatings are used, mainly waxes and acrylic resins, as they comply with the requirements of conservation ethics [2]. However, the drawbacks of these coatings are a reduced protection efficiency and short lifetime in aggressive environments such as the urban atmosphere [2,3]. Although new types of coatings, such as organosilanes [4,5], carboxylates [6,7], fluoropolymers [8,9], and polyurethanes [10], have been developed in recent decades, waxes and acrylic coatings are still preferred by conservators. In order to increase their protection efficiency and durability, these coatings are often combined with corrosion inhibitors. An example is Incralac, a commercial acrylic coating containing benzotriazole (BTA) as a corrosion inhibitor and UV stabilizer. Considering the prevalence of Incralac in conservation practice, new environmentally friendly and non-toxic corrosion inhibitors are being investigated with the aim of replacing the harmful BTA [11,12]. Today, despite the great efforts to develop a durable protection for our bronze cultural heritage, this is still an unsolved problem.

Adhesion is the one of the most important properties of any coating as it affects its protection efficiency and durability. Thus, by improving the coating adhesion, its performance can be enhanced. One of the ways to achieve this is to use molecular promoters of coating adhesion. In order to act as an adhesion promoter, a molecule should be bifunctional. This means that it must contain a functional group that can bind to the substrate (head group) and another functional group that can react with the coating (terminal group). Most studies with molecular promoters have been carried out on aluminum [13,14,15,16] and mild steel [17,18,19], while there is a little work on copper [20,21] and bronze [22,23]. The most studied group of molecules, able to create stronger interfacial interaction between the coating and the substrate, are organosilanes [19,24]. They can be used as coating additives or as primers. Aminealkylthiols have also been shown to improve the adhesion of epoxy coating to copper [21]. A covalent bond is formed between the thiol functional group and copper [25,26], while amine reacts with the epoxy coating. Phosphonic acids are very promising adhesion promoters as they have a strong binding affinity to a variety of surfaces and can form stable films [27,28,29]. They have already proven to be good corrosion inhibitors for copper and its alloys, as they form an effective barrier against the penetration of corrosive and oxidizing chemicals towards the substrate surface [30,31,32]. The terminal functional group of such compounds is important because it determines the physical and chemical properties of the modified substrate. Kosec et al. [33] were able to create hydrophobic surfaces of copper and bronze with myristic acid due to its terminal methyl group. In the work of Denayer et al. [21], the presence of a terminal methyl group on copper led to the displacement of the epoxy from the surface due to hydrophobic–hydrophilic repulsion, while the terminal amine group improved its adhesion. The adhesion of the epoxy coating was also successfully increased with 4-amino-butyl-phosphonic acid on carbon steel [34].

Improving the efficiency and durability of a coating has multiple benefits. The number of conservation treatments is minimized, which means that less time and money are spent and also that the sculpture to be conserved will suffer less damage. As the original appearance of the sculpture must be retained, the compounds applied to improve adhesion should also be carefully selected in order to avoid changes in the color of the substrate [35].

The aim of this work was to examine two phosphonic acids with different end groups, 16-phosphonohexadecanoic acid (COOH-PA) and 12-aminododecylphosphonic acid (NH_2_-PA), as possible corrosion inhibitors and adhesion promoters of polyurethane and Paraloid B72 coatings on bronze. In our previous work, it was found that COOH-PA improved the protection efficiency and durability of Paraloid B72 on patinated bronze, but the influence on coating adhesion could not be determined due to the low adhesion of patina [22]. Similarly, NH_2_-PA demonstrated a beneficial effect on the protective properties of waterborne acrylic coating on bare and patinated bronze [23]. Thus, pretreatment of bronze with selected phosphonic acids presents a promising solution for enhancement of the clear coating protection of bronze. In order to gain a deeper insight into the influence of the mentioned phosphonic acids on polyurethane coating and Paraloid B72 performance in bronze protection, their protective effect and durability were examined in this work by electrochemical measurements, while their influence on coating adhesion was examined by pull-off test.

## 2. Materials and Methods

### 2.1. Materials

Studies were conducted on bronze samples cut from CuSn12 bronze rod (composition given in Table 1) such that their thickness was 0.5 cm and the surface area 1.33 cm^2^. Bronze was obtained from Strojopromet Ltd., Zagreb, Croatia. A copper wire was soldered on the back side of the bronze disks, and the samples were then embedded into the epoxy resin, exposing an electrode surface area of 1.33 cm^2^.

16-phosphonohexadecanoic acid (97%, Sigma Aldrich Corp., Saint Louis, MO, USA) (COOH-PA) and 12-aminododecylphosphonic acid (95%, Alfa Aesar, Karlsruhe, Germany) (NH_2_-PA) were dissolved in ethanol (96% p.a., Lach-ner d.o.o., Zagreb, Croatia) to form 1 mM solutions and used to create protective films on bronze. Paraloid B72 (C.T.S., Altavilla Vicentina, Italy) dissolved in ethyl acetate (p.a., T.T.T. d.o.o., Sveta Nedjelja, Croatia), and polyurethane (Draco, Solin, Croatia) were used for bronze coating. Artificial acid rain was prepared by dissolving 0.2 g/L NaNO_3_ (p.a., T.T.T., Sveta Nedelja, Croatia), 0.2 g/L NaHCO_3_ (p.a., Kemika, Zagreb, Croatia), and 0.2 g/L Na_2_SO_4_ (p.a., Kemika, Zagreb, Croatia) in deionized water. The pH of the obtained artificial rain was adjusted to 5 with 0.5 M H_2_SO_4_. The conductivity of the artificial rain solution was 0.56 mS cm^−1^.

### 2.2. Sample Preparation

Bronze coupons were ground with 800-, 1200-, and 2500-grit SiC papers, polished with α-Al_2_O_3_ (particle size 0.1 μm), degreased with ethanol in an ultrasonic bath, and rinsed with deionized water.

Deposition of COOH-PA and NH_2_-PA films on bronze surface was performed in three steps established in our previous studies [31,36,37]. The first step included oxide formation in an air convection oven for 24 h at 80 °C. The following step was adsorption of phosphonic acids through immersion of oxidized bronze samples in COOH-PA and NH_2_-PA ethanolic solutions solution for 20 h at 40 °C. In the final step, the samples were dried for 5 h at 80 °C. As such procedure results in the formation of organic films with multilayer structures [32], this work additionally examined simple methods for removing upper layers of the film in order to obtain COOH-PA and NH_2_-PA monolayers. These studies were conducted on COOH-PA films, and the most suitable method was also applied to remove the upper layers of NH_2_-PA film.

The first method of monolayer preparation (Ultrasonic bath 1) assessed the removal of upper layers by placing samples in ethanol and in an ultrasonic bath for 30 min, immediately after the adsorption step. Samples were then dried for 5 h at 80 °C. The second approach (Ultrasonic bath 2) used the same procedure but after the third step (drying films for 5 h at 80 °C). Other examined methods included rinsing with ethanol for 10 s (Rinsing) or wiping upper layers of the film with lens cleaning tissue soaked in ethanol (Wiping) after the drying step. Additional drying for 30 min at 80 °C was applied for the last three methods. The purpose of the drying step was to stimulate the reorganization of molecules in the film upon the removal of upper layers. A description of each method is presented in Table 2.

Paraloid B72 (15% *w*/*v* solution in ethyl acetate coatings) and polyurethane coating were applied with a brush either on bare bronze samples or those covered by phosphonic acid film. Coatings were applied in only one layer in order to see the effect of the phosphonic acid films on the protective properties of the coating in the shortest possible time. After the coating application, samples were dried at room temperature for three days. The dry film thickness measured with PosiTector 6000 (DeFelsko, Ogdensburg, NY, USA) for Paraloid B72 was 12 ± 4 μm, and for polyurethane it was 20 ± 3 μm (determined in 10 measurement points for each coating). Although coatings were applied manually, satisfactory reproducibility of coating thickness was achieved. Surface pretreatments by phosphonic acids did not influence the overall coating thickness, as the film thickness was only a few nm [32].

### 2.3. Electrochemical Measurements

Corrosion behavior of studied samples was examined by electrochemical methods during the immersion in acid rain solution for three weeks. Some samples were also exposed to an outdoor urban atmosphere for three weeks with initial and final electrochemical characterization in an artificial rain solution. Samples were placed outdoors, close to a busy road in the center of the city of Zagreb. Based on the relevant meteorological data [38], the corrosivity of the atmosphere was estimated according to the EN ISO 9223 standard [39], which corresponds to the C2 category for copper.

Initial electrochemical measurements were performed after the sample had been immersed in the corrosive medium for 45 min to avoid a change in the open circuit potential (*E*_ocp_) during the acquisition of polarization or impedance data. The period of 45 min was sufficiently long for all studied samples to reach a steady state.

The electrochemical measurements were performed using a Bio-Logic SP-300 potentiostat and were carried out in a three-electrode cell. The bronze sample was set as the working electrode, while a saturated calomel electrode (SCE) and a Pt plate were used as the reference and counter electrodes, respectively. Linear polarization measurements were performed in a narrow (±20 mV vs. *E*_ocp_) potential range at the scan rate of 0.166 mV s^−1^. Electrochemical impedance spectroscopy (EIS) measurements were conducted at *E*_ocp_ in the frequency range from 100 kHz to 10 mHz with a 10 mV amplitude. At least three replicas of each type of sample were used for electrochemical measurements.

### 2.4. AFM Measurements

In order to investigate the morphology and calculate the surface roughness of blank bronze substrate and those modified by phosphonic acid films, atomic force microscopy was performed on the Nanosurf Core AFM (Nanosurf, Liestal, Switzerland). The measurements were performed in the tapping (dynamic) mode using a tip with a 40 Nm^−1^ force constant. The images were processed and analyzed in the Nanosurf C3000 v3.10.5 software using the line fit. Calculated root mean square roughness parameters *S*_q_ and *R*_q_ were obtained from the 5 × 5 μm area and as an average of 10 cross-section cuts, respectively.

### 2.5. Pull-Off Test

The pull-off test was used to measure the adhesion of coatings to a bare bronze substrate and to bronze substrates pretreated with phosphonic acids. An automatic adhesion tester DeFelsko PosiTest AT-A was used. The dolly (pull stub) was abraded and degreased in ethanol. After that, two-component resin was added to the dolly, which was then adhered to the coated bronze surface. Resin was cured for 24 h before performing the test. At least four measurements were taken per sample.

## 3. Results and Discussion

### 3.1. Monolayer Preparation

In our previous studies, it was found that self-assembled films of phosphonic acids can provide corrosion protection to copper alloys [31,32]. In general, it was observed that the thicker the organic film, the better was the corrosion protection, and for that reason, films with multilayer structures were formed. However, when phosphonic acids are used as molecular promoters of coating adhesion, monolayer films are desirable. The intermolecular bonds between the upper layers of the film are significantly weaker compared to the bond between the bronze surface and the chemisorbed first layer of phosphonic acid [40]. If the coating is applied on multilayered film, weak interaction between layers can result in low coating adhesion.

Although tetrahydrofuran (THF) is mostly used as a solvent of long-chain phosphonic acids in the case of monolayer preparation [41,42,43,44,45], in this work, ethanol was chosen as the more environment friendly solvent. However, films formed from ethanol usually have a multilayer structure, and for that reason, different methods of removing upper layers of the film were investigated. In principle, it is possible to control the number of upper layers by decreasing the adsorption duration or phosphonic acid solution concentration, but our previous research [32] showed that in such cases, it is possible that the obtained films are not well-ordered, which will negatively impact their performance. It is expected that upper layers of a phosphonic acid multilayer film can easily be removed from the surface by chemical or physical methods, as such films are not chemically bound to the surface. On the other hand, the first chemisorbed layer should not be affected by such procedures. In this work, simple methods for their removal were chosen, such as rinsing or cleaning with ethanol. A description of each examined method is presented in Table 2.

In order to gain an insight into the stability of films after the removal of upper layers, linear polarization measurements were conducted, from which polarization resistance values (*R*_p_) were determined. Although the initial condition of the film is the most important for improving the coating adhesion, its corrosion protection efficiency can be an indication of film crystallinity and strong attachment to the surface. It is preferable for the film to be well organized and evenly distributed over the surface, with few defects in the film before coating application.

**Figure 1:**
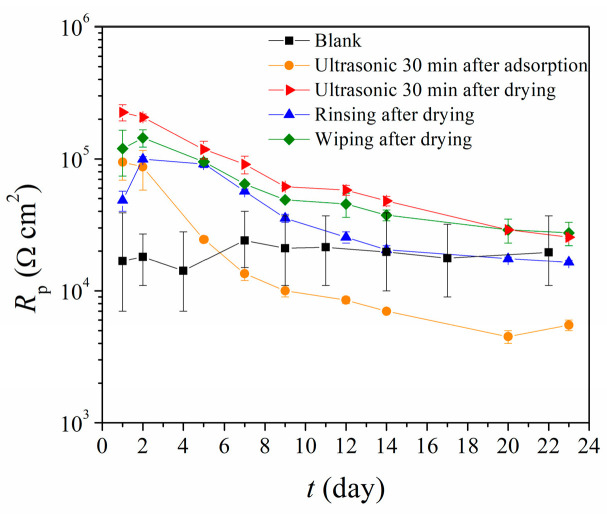
Polarization resistance (*R*_p_) as a function of immersion time in simulated acid rain for bare bronze (blank) and samples with differently prepared phosphonic acid films.

The film that was in the ultrasonic bath for 30 min, immediately after the adsorption (Ultrasonic bath 1), provided the weakest protection (Figure 1). Although its initial *R*_p_ was higher than that of the blank sample, it decreased rapidly in time and, after one week, became lower than that of the blank sample. By using an ultrasonic bath after the drying step, significantly better results over time were obtained. This indicates the importance of the drying step before any removal of the upper layers of the film, as drying enhances the chemisorption of molecules on the surface and increases the stability of the first monolayer [36,46].

Rinsing with ethanol also resulted in films with poor protective properties. When the film is continuously exposed to the stream of ethanol for ten seconds, it is possible that the film dissolves unevenly, causing defects in its structure. The wiping after drying results in the film with the lowest decrease in *R*_p_ value over time, which leads to the conclusion that such a film is the most stable, which could offer an indication that only a chemisorbed monolayer remains on the bronze surface.

**Figure 2:**
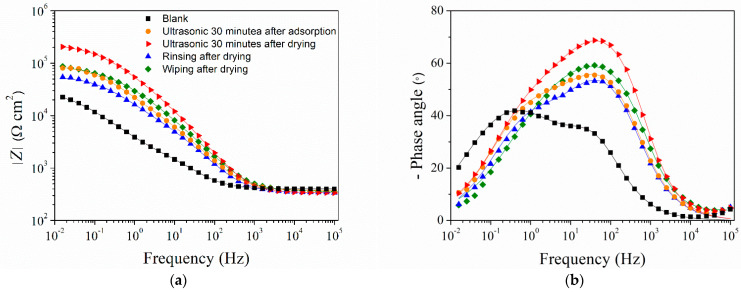
EIS spectra (**a**) impedance modulus and (**b**) phase angle Bode plot for bare bronze (blank) and samples with differently prepared COOH-PA films. Symbols: experimental data; solid lines: modeled data according to electrical equivalent circuits given in Figure 3.

**Figure 3:**
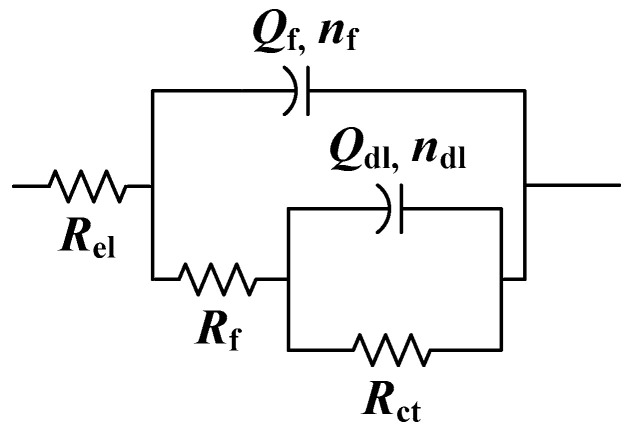
Selected electrical equivalent circuit.

Aside from monitoring the *R*_p_ evolution over time, EIS measurements were also conducted immediately after monolayer formation. The obtained EIS spectra (Figure 2) show a similar trend of impedance modulus values at the lowest frequencies, as observed from linear polarization measurement, while the phase angle plots are characterized by two phase angle maxima for all samples. For this reason, EIS spectra were modeled with electrical equivalent circuits (EECs) with two time constants (Figure 3).

Due to the inhomogeneity of the studied surfaces, the capacitor element in electrical equivalent circuits was replaced with a constant-phase element (CPE). The impedance of the CPE is defined as *Z*_CPE_ = 1/[Q(jω)^n^], where *Q* is the CPE’s frequency-independent parameter, which represents pure capacitance when *n* = 1. The first couple in the EEC is *R*_f_ − *Q*_f_, where *R*_f_ represents the resistance of the surface film, which is a composite of oxides and phosphonic acid film (as phosphonic acids adsorb on oxides), while *Q*_f_ is related to its capacitance. Similar was observed by other authors, studying protective organic films on oxidized metal surfaces [47,48]. The second couple, *R*_ct_ − *Q*_dl_, describes the corrosion reaction at the metal substrate/solution interface, where *R*_ct_ is charge transfer resistance and *Q*_dl_ is the constant-phase element representing the double-layer capacitance. The electrolyte resistance between the working and reference electrodes is represented by *R*_el_. The spectra for a blank sample can be fitted to the same EEC, but the *R*_f_ − *Q*_f_, couple then corresponds to an oxide layer.

The results of regression calculation are given in Table 3. The most protective films (highest *R*_f_ values) were obtained for samples from the ultrasonic bath treatment after drying and for wiped samples. However, if pseudocapacitance values (*C*_f_) are calculated from the *Q*_f_ values of these samples, it appears that the *C*_f_ value for a wiped sample is twice that of a sample placed in an ultrasonic bath after drying. Thus, the COOH-PA film of the latter sample is twice the thickness of the film on the wiped sample, i.e., it is not a monolayer film. Another important parameter in the film quality evaluation is *Q*_dl_. Adsorption of organic molecules on bronze decreased the amount of water at the metal/solution interface, and thus lower *Q*_dl_ values were observed. The lowest *Q*_dl_ were obtained for samples ultrasonically cleaned after drying and for wiped samples, together with relatively high *R*_ct_ values, so it can be assumed that for these samples, the phosphonic acid film densely covers the bronze surface, creating a barrier to electrolyte penetration. These results confirm that COOH-PA acts as a bronze corrosion inhibitor in the studied conditions. Taking into account these results, the wiping method appears to be the best method for obtaining thin and stable phosphonic acid films on bronze, and it was selected as a simple and practical method for surface pretreatment before applying organic coatings.

**Figure 4:**
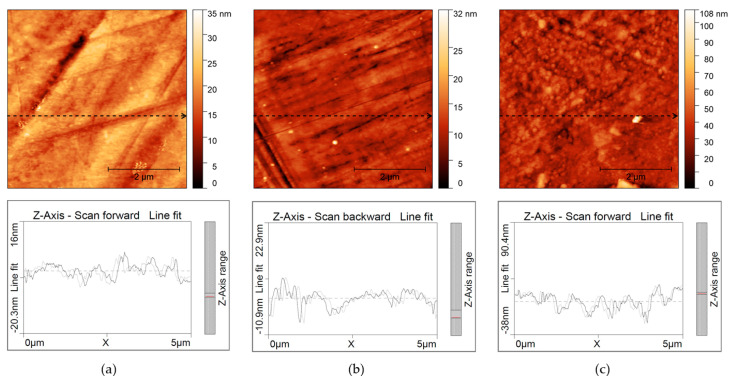
AFM images of (**a**) blank bronze, (**b**) bronze with COOH-PA film formed by wiping procedure, (**c**) bronze with COOH-PA film prior to wiping procedure.

In order to confirm that the obtained film had a monolayer structure, AFM measurements were conducted. It is usually considered that if the roughness of a modified electrode is not significantly different from that of the modified surface, then a monolayer film is formed [49,50]. Figure 4 presents AFM images of a blank bronze surface and one threated with COOH-PA, before and after a wiping procedure. It can be observed that on the blank sample (Figure 4a). polishing lines are visible, which cannot be seen on the sample with a PA film, without wiping (Figure 4c); this indicates the presence of a multilayer film. After wiping, the morphology of the surface (Figure 4b) becomes similar to that of the blank sample. This is also confirmed from the values of roughness parameters presented in Table 4, showing that the roughness levels of blank and wiped samples are similar.

### 3.2. Polyurethane Coating

**Figure 5:**
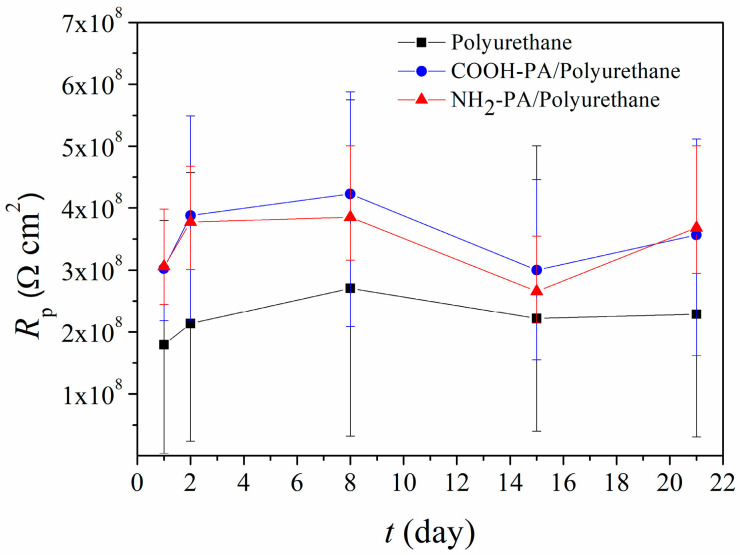
Polarization resistance as a function of immersion time in simulated acid rain for bronze protected with polyurethane coating with and without COOH-PA and NH_2_-PA pretreatment.

After the method for the preparation of phosphonic acid films was established, their influence on the protective properties of two organic coatings was examined. Firstly, studies were conducted with a clear polyurethane coating. Two long-chain phosphonic acids (COOH-PA and NH_2_-PA) were examined in combination with the polyurethane coating. Polarization measurements were conducted on samples protected by the coating, with or without surface pretreatment with phosphonic acids. The evolution of polarization resistance values during a two-week exposure to acid rain solution is presented in Figure 5. This indicates that both COOH-PA and NH_2_-PA improve the protective properties of polyurethane coating. However, this improvement is moderate (two-fold) for both acids.

**Figure 6:**
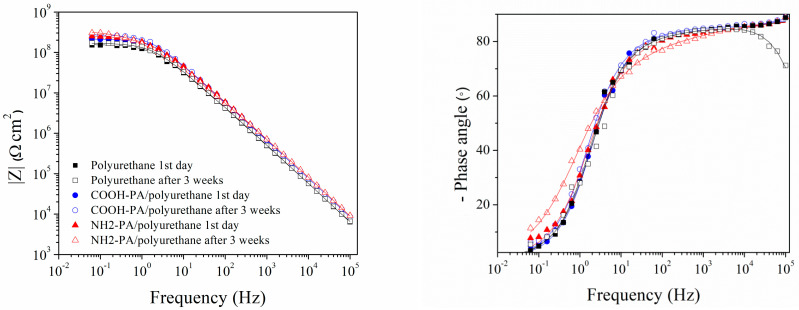
EIS—Bode plot for the bronze protected with polyurethane coating with and without COOH-PA and NH_2_-PA pretreatment exposed to acid rain. Symbols: experimental data; solid lines: modeled data according to electrical equivalent circuit given in Figure 7.

In addition to polarization measurements, samples were also characterized by EIS. The EIS spectra, obtained on the first day of immersion and after three weeks, are given in Figure 6. The impedance modulus values, at the lowest frequencies, of phosphonic-acid-pretreated samples are higher compared to samples protected only with polyurethane both at the beginning and at the end of immersion.

**Figure 7:**
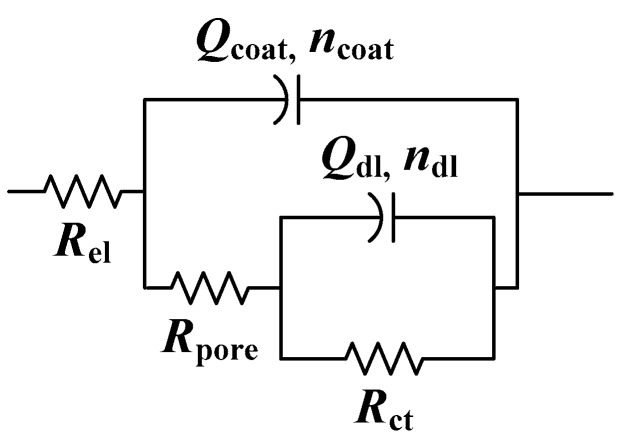
Selected electrical equivalent circuit for bronze protected with coating.

Two poorly resolved phase angle maxima were observed in the high- and medium-frequency regions for all samples. Therefore, the EIS results were analyzed with EEC with two time constants (Figure 7). The first time constant consists of the pore resistance of the coating, *R*_pore_, and coating capacitance, *Q*_coat_. The second time constant is *R*_ct_ − *Q*_dl_ [22,50,51]. In Table 5 are the results of regression calculation. The high values of *R*_pore_ and *n*_coat_ for samples protected with phosphonic acids and the coating, compared to the sample where only coating was applied, confirm the beneficial influence of both phosphonic acids in improving the protective properties of polyurethane. *R*_ct_ is also higher for these samples, which indicates a lower dissolution of the bronze substrate compared to the sample protected only with polyurethane. The positive effect of treating the bronze surface with phosphonic acids is relatively moderate, which is probably due to the excellent protective properties of polyurethane coating alone.

In order to determine the effect of COOH-PA and NH_2_-PA on the adhesion of the polyurethane coating to the bronze substrate, pull-off adhesion testing was performed. The results presented in Table 6 clearly show that both COOH-PA and NH_2_-PA increased the coating adhesion, where the stronger improvement is observed for NH_2_-PA.

### 3.3. Paraloid B72 in Acid Rain

**Figure 8:**
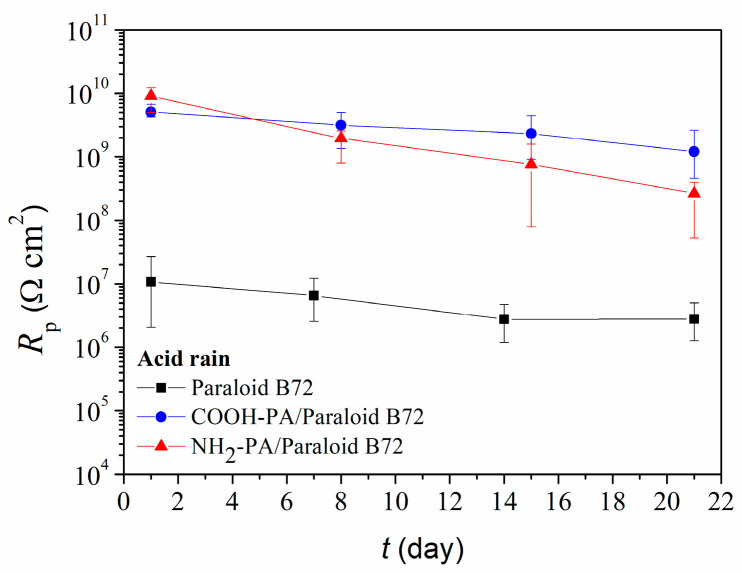
Polarization resistance as a function of immersion time in simulated acid rain, for bronze protected with Paraloid B72 alone or in combination with COOH-PA or NH_2_-PA.

Further studies were conducted with acrylic coating Paraloid B72. Corrosion protection offered by the coating alone and by a combined protective system of phosphonic acid/coating was examined during three weeks of exposure to simulated acid rain solution. A significant improvement in the protective properties of Paraloid B72 upon the surface pretreatment with both studied phosphonic acids was obtained (Figure 8). Polarization resistance values, determined for pretreated samples, were two orders of magnitude higher than those obtained for coated-only samples. Initially, the NH_2_-PA acid in combination with Paraloid B72 gave better results, while after three weeks in acid rain, COOH-PA showed slightly better properties.

**Figure 9:**
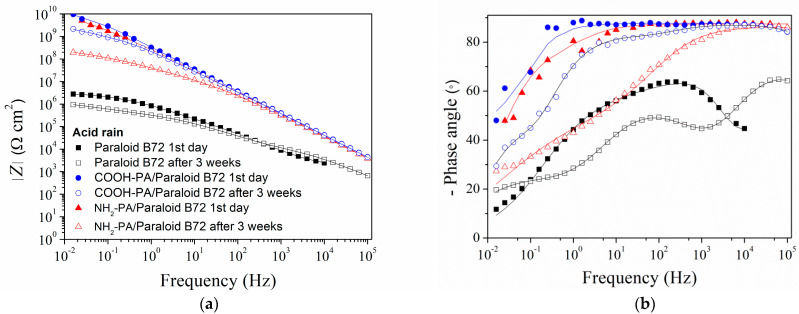
EIS spectra: (**a**) impedance modulus and (**b**) phase angle Bode plot for the bronze protected with Paraloid B72 with and without COOH-PA and NH_2_-PA pretreatment exposed to acid rain. Symbols: experimental data; solid lines: modeled data according to electrical equivalent circuits given in Figure 7 and Figure 10.

With the aim of better understanding the influence of phosphonic acid pretreatment on the protective properties of Paraloid B72, EIS measurements were conducted. From the impedance modulus plot (Figure 9a), it can be seen that both COOH-PA and NH_2_-PA significantly contribute to the protective properties of Paraloid B72 in artificial acid rain, as the impedance values in the whole measured frequency region are significantly higher. The phase angle plots for pretreated samples exhibited wide maxima close to −90°, which is typical of coatings with excellent barrier properties. After three weeks, the impedance modulus decreased, followed by a change in the phase angle plot. However, the decrease in the impedance of samples pretreated with COOH-PA was less pronounced than in the case of NH_2_-PA-pretreated samples.

**Figure 10:**
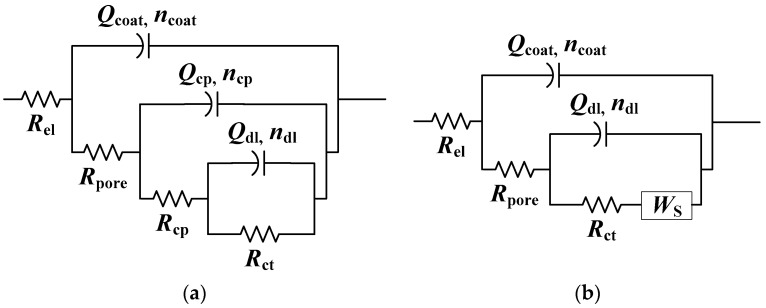
Selected electrical equivalent circuits for (**a**) sample with B72 coating after 3 weeks and (**b**) sample with COOH-PA/B72 coating after 3 weeks.

For the first day of immersion in corrosive medium, the EIS spectra are characterized with two phase angle maxima. Therefore, the previously described EEC with two time constants was used for all samples (Figure 7). The same EEC was used to fit EIS spectra of the NH_2_-PA-pretreated sample after three weeks of immersion in acid rain solution. In the case of the COOH-PA-pretreated sample and the reference sample with Paraloid B72, the complexity of the spectra required the use of a more complex EEC (Figure 10). For the sample without pretreatment, third-phase angle maxima occurrence was attributed to the formation of reactive corrosion products with faradaic resistance *R*_cp_ and capacitance *Q*_cp_ [52,53]. The EIS spectra for the sample pretreated with COOH-PA after three weeks in a corrosive medium were better fitted with the EEC containing a finite Warburg element (*W*_s_) (Figure 10b), which describes the finite length diffusion in pores of the coating [54].

The results of regression calculation are given in Table 7. *R*_pore_ is significantly higher for samples pretreated with COOH-PA and NH_2_-PA. It can be assumed that molecules of phosphonic acids are filling the pores of the coating, slowing the penetration of the electrolyte towards the bronze surface. After three weeks in acid rain solution, the *R*_pore_ of these samples decreased due to the electrolyte penetration, which can be seen from the increase in *Q*_coat_ and *Q*_dl_ [55,56]. However, the protective properties of the coating combined with a phosphonic acid film are significantly better than for the coating alone, even after three weeks in a corrosive medium. The fact that the *R*_ct_ value is significantly higher for pretreated samples indicates that both phosphonic acids inhibit the dissolution of bronze.

Further evaluation of the influence of phosphonic acid pretreatment on Paraloid B72 performance was conducted through the exposure of coated-only and COOH-PA-pretreated samples to an outdoor urban atmosphere. COOH-PA pretreatment was selected as it showed a better performance than NH2-PA over a longer immersion period.

### 3.4. Paraloid B72 Outdoor Exposure

**Figure 11:**
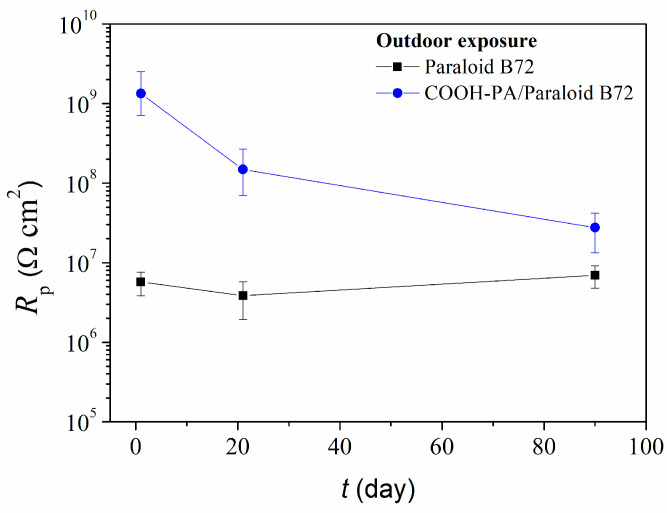
Polarization resistance as a function of outdoor exposure time for bronze protected with Paraloid B72 alone or in combination with COOH-PA.

As for the studies conducted in acid rain solution, COOH-PA significantly contributed to the protection properties of Paraloid B72 exposed to the outdoor atmosphere (Figure 11). After three weeks of exposure, the *R*_p_ value of the combined protection system decreased, but was still one order of magnitude higher than the *R*_p_ of the coating alone.

**Figure 12:**
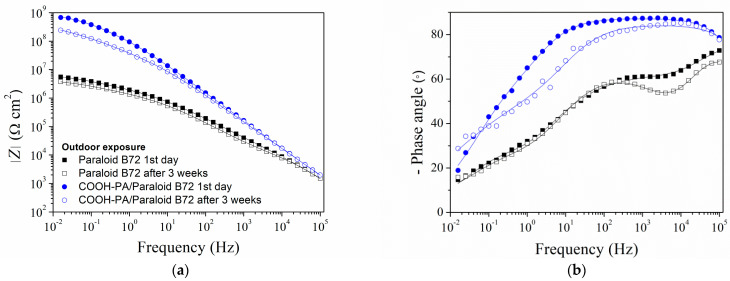
EIS spectra: (**a**) impedance modulus and (**b**) phase angle Bode plot for the bronze protected with Paraloid B72 alone or in combination with COOH-PA exposed to the outdoor atmosphere. Symbols: experimental data; solid lines: modeled data according to electrical equivalent circuits given in Figure 3.

EIS measurements were conducted on samples exposed to the outdoors (initial characterization and after three weeks), and the results are presented in Figure 12. High phase angle values in a wide range of frequencies before and after exposure to the outdoor atmosphere for samples protected with COOH-PA/Paraloid B72 show good barrier properties of this protection system. A decrease in the impedance modulus, in the low-frequency region, over time can be observed, as in the case of samples immersed in acid rain solution, but the change in the phase angle is much smaller. This indicates lower degradation of Paraloid B72 when exposed to the outdoors.

As most of the EIS spectra exhibited two phase angle maxima, they were fitted with the previously described EEC with two phase angle maxima (Figure 7). Only for EIS spectra collected for B72 after 3 weeks of immersion was a more complex model needed (Figure 10a). Both the pore resistance and charge transfer resistance values were significantly lower for the sample protected only with Paraloid B72 compared to that with COOH-PA/Paraloid B72 (Table 8). This difference was significant even after the exposure to outdoor conditions. After three weeks, an increase in *R*_pore_ for the Paraloid B72 sample was observed, which is related to the formation of corrosion products that clog the pores of the coating.

For pretreated samples, high *n*_coat_ values are an indication of their compactness and homogeneity, while small changes in the EIS parameters over time indicate that the coating retains its initial properties.

In order to determine the influence of COOH-PA and NH_2_-PA pretreatment on Paraloid B72 coating adhesion, pull-off adhesion testing was performed. For sample without phosphonic acid pretreatment, the pull-off strength was around 5.51 MPa. The samples treated with COOH-PA and NH_2_-PA came to glue failure at forces of 8.03 and 7.96, respectively (Table 9). This indicates stronger adhesion of the coating to the bronze substrate when the surface is pretreated with phosphonic acids.

## 4. Conclusions

The aim of this work was to examine whether the corrosion protection of bronze by two clear coatings could be enhanced by surface modification with monolayers of two phosphonic acids. In addition, their performance as coating adhesion promoters was investigated.

Electrochemical studies of the corrosion behavior of bronze protected by a polyurethane coating showed that both NH_2_-PA and COOH-PA increase the level of corrosion protection offered by the coating, as was observed throughout the three-week immersion of samples in acid rain solution. In addition, both phosphonic acids increased coating adhesion, with slightly better results for NH_2_-PA.

In the case of the acrylic coating Paraloid B72, the enhancement of corrosion protection by the studied phosphonic acids was even more pronounced than in the case of the polyurethane coating. Initially, NH_2_-PA gave superior results, while after three weeks in acid rain, COOH-PA showed slightly better properties. Additional studies, conducted on samples exposed to the outdoor atmosphere, confirmed the beneficiary effect of COOH-PA pretreatment. Furthermore, both acids significantly improved coating adhesion.

The results obtained in this study clearly confirm that both studied phosphonic acids can be used as corrosion inhibitors and coating adhesion promoters, thus providing a simple solution for improved durability and protection of polyurethane and acrylic coatings on bronze surfaces.

## Figures and Tables

**Table 1:** Compositions of studied CuSn12 bronze.

**CuSn12**	**Element**	**Cu**	**Sn**	**Pb**	**Ni**	**P**	**Zn**	**Rest**
	Wt. (%)	87.94	11.02	0.54	0.29	0.10	0.07	0.04

**Table 2:** Methods of phosphonic acid monolayer preparation.

Method	Steps				
	1	2	3	4	5
**Ultrasonic bath 1**	**Bronze oxidation** 24 h at 80 °C	**Film adsorption** 20 h at 40 °C	30 min in ethanol in **ultrasonic bath**	**Film drying** 5 h at 80 °C	/
**Ultrasonic bath 2**			**Film drying** 5 h at 80 °C	30 min in ethanol in **ultrasonic bath**	**Film drying** 30 min at 80 °C
**Rinsing**				**Rinsing** under a stream of ethanol for 10 s	
**Wiping**				**Wiping** with lens tissue soaked in ethanol	

**Table 3:** EIS parameters for samples after applying different methods for removing upper layers of COOH-PA film, obtained by fitting experimental data to selected equivalent circuit (Figure 3).

Method	*R*_f_ (kΩ cm^2^)	*Q*_f_ (µS s^n^ cm^−2^)	*n*_f_	*R*_ct_ (kΩ cm^2^)	*Q*_dl_ (µS s^n^ cm^−2^)	*n*_dl_
Ultrasonic: 30 min after adsorption	13	4.88	0.79	80	9.74	0.64
Ultrasonic: 30 min after drying	32	1.91	0.88	203	4.34	0.59
Rinsing	9.6	5.77	0.79	48	15.32	0.65
Wiping	22	3.94	0.79	65	8.69	0.67

**Table 4:** Calculated roughness parameters *S*_q_ and *R*_q_ for blank and PA-treated samples.

Sample	*R*_q_ (nm)	*S*_q_ (nm)
Blank	2.3	3.5
COOH-PA before wiping	11.5	10.0
COOH-PA after wiping	2.8	2.3

**Table 5:** EIS parameters for the bronze protected with polyurethane coating with and without COOH-PA and NH2-PA pretreatment exposed to outdoor atmosphere obtained by fitting experimental data to selected equivalent circuit (Figure 6).

	*R*_pore_ (kΩ cm^2^)	*Q*_coat_ (nS s^n^ cm^−2^)	*n*_coat_	*R*_ct_ (MΩ cm^2^)	*Q*_dl_ (nS s^n^ cm^−2^)	*n*_dl_
	Polyurethane					
1st day	473	0.3	0.98	157	0.5	0.74
After 3 weeks	2.44	0.5	0.79	170	0.3	0.99
	COOH-PA/Polyurethane					
1st day	5000	0.2	0.98	215	0.3	0.77
After 3 weeks	2301	0.2	1.00	275	0.4	0.77
	NH_2_-PA/Polyurethane					
1st day	4727	0.2	0.98	256	0.5	0.70
After 3 weeks	1584	0.3	0.97	349	1	0.62

**Table 6:** Pull-off strength between polyurethane coating and CuSn12 bronze with and without pretreatment with phosphonic acids.

System	Pull-Off Strength (MPa)
CuSn12/Polyurethane	7.54 ± 0.33
CuSn12/COOH-PA/Polyurethane	8.13 ± 0.17
CuSn12/NH_2_-PA/Polyurethane	8.60 ± 0.21

**Table 7:** EIS parameters for the bronze protected with Paraloid B72 with and without COOH-PA and NH2-PA pretreatment exposed to acid rain, obtained by fitting experimental data to selected equivalent circuits (Figure 7 and Figure 10).

	**B72**								
	*R*_pore_ (MΩ cm^2^)	*Q*_coat_ (nS s^n^ cm^−2^)	*n*_coat_	*R*_cp_ (MΩ cm^2^)	*Q*_cp_ (µS s^n^ cm^−2^)	*n*_cp_	*R*_ct_ (MΩ cm^2^)	*Q*_dl_ (µS s^n^ cm^−2^)	*n*_dl_
1st day	0.007	290	0.62	-	-	-	3.1	0.001	0.95
After 3 weeks	0.011	3	0.81	0.352	0.36	0.65	1.3	2.78	0.50
	**COOH-PA/B72**								
	*R*_pore_ (MΩ cm^2^)	*Q*_coat_ (nS s^n^ cm^−2^)	*n*_coat_	*R*_ct_ (MΩ cm^2^)	*Q*_dl_ (µS s^n^ cm^−2^)	*n*_dl_	*Coth Yo (nS* s^0.5^ cm^−2^*)*	*B* (s^0.5^*)*	
1st day	8300	0.5	0.97	1.3 × 10^4^	0.001	1.00	-	-	
After 3 weeks	18	0.5	0.97	966	3 × 10^−4^	0.73	2.2	4.3	
	**NH_2_-PA/B72**								
	*R*_pore_ (MΩ cm^2^)	*Q*_coat_ (nS s^n^ cm^−2^)	*n*_coat_	*R*_ct_ (MΩ cm^2^)	*Q*_dl_ (µS s^n^ cm^−2^)	*n*_dl_			
1st day	810	0.6	0.81	8930	3 × 10^−4^	0.65			
After 3 weeks	320	0.7	0.96	350	8 × 10^−3^	0.50			

**Table 8:** EIS parameters for the bronze protected with Paraloid B72 with and without COOH-PA and NH_2_-PA pretreatment exposed to the outdoor atmosphere, obtained by fitting experimental data to selected equivalent circuits (Figure 7 and Figure 10).

	*R*_pore_ (MΩ cm^2^)	*Q*_coat_ (nS s^n^ cm^−2^)	*n*_coat_	*R*_cp_ (MΩ cm^2^)	*Q*_cp_ (nS s^n^ cm^−2^)	*n*_cp_	*R*_ct_ (MΩ cm^2^)	*Q*_dl_ (nS s^n^ cm^−2^)	*n*_ct_
	**B72**								
1st day	0.006	150	0.50	-	-	-	6.55	4.01	0.92
After 3 weeks	0.029	50	0.71	1.63	5	0.70	7.60	350	0.56
	COOH-PA/B72								
1st day	100	1.26	0.97	-	-	-	910	2.37	0.59
After 3 weeks	12	1.88	0.94	-	-	-	540	8.23	0.50

**Table 9:** Pull-off strength between Paraloid B72 and CuSn12 bronze with and without pretreatment with COOH-PA and NH_2_-PA.

System	Pull-Off Strength (MPa)
CuSn12/Paraloid B72	5.51 ± 1.13
CuSn12/COOH-PA/Paraloid B72	8.03 ± 0.34
CuSn12/NH_2_-PA/Paraloid B72	7.96 ± 2.10

## Data Availability

Data that support the findings of this study are available from the corresponding author upon reasonable request.
